# Re-irradiation for locoregionally recurrent tumors of the thorax: a single-institution, retrospective study

**DOI:** 10.1186/s13014-016-0673-z

**Published:** 2016-08-02

**Authors:** Kiyomi Sumita, Hideyuki Harada, Hirofumi Asakura, Hirofumi Ogawa, Tsuyoshi Onoe, Shigeyuki Murayama, Satoaki Nakamura, Noboru Tanigawa, Toshiaki Takahashi, Tetsuo Nishimura

**Affiliations:** 1 Radiation and Proton Therapy Center, Shizuoka Cancer Center, 1007 Shimonagakubo, Nagaizumi-cho, Sunto-gun, Shizuoka, 411-8777 Japan; 2 Department of Radiology, Kansai Medical University, 2-5-1 Shinmachi, Hirakatashi, Osaka, 573-1010 Japan; 3 Division of Thoracic Oncology, Shizuoka Cancer Center, 1007 Shimonagakubo, Nagaizumi-cho, Sunto-gun, Shizuoka, 411-8777 Japan

**Keywords:** Lung cancer, Recurrence tumor, Re-irradiation

## Abstract

**Background:**

Re-irradiation (re-RT) of the thorax is challenging due to the impact of prior therapies on normal tissues, and there are few reports of definitive re-RT. The treatment toxicities and efficacy of re-RT are not well known. The aim of the present study was to assess the safety and efficacy of definitive re-RT of the thorax.

**Methods:**

Patients who were treated with thoracic re-RT between March 2007 and December 2014 were retrospectively analyzed. Primary and re-irradiation plans were required to have an overlap of dose distributions for the 80 % isodose level. All doses were recalculated to an equivalent dose of 2 Gy per fraction (EQD2). When possible, analysis of dose accumulation was carried out using the medical image merge (MIM) (®) software program (version 6.5, MIM Software Inc., Cleveland, OH). Administration dosages for organs at risk were defined.

**Results:**

Fourteen (67 %) and seven (33 %) patients with non-small cell carcinoma (NSCLC) and small cell carcinoma (SCLC), respectively, were identified. The patients’ median age was 72 (range 53–85) years. Fifteen patients (71 %) had “proximal” tumors, defined as tumors at the distal 2 cm of the trachea, carina, and main bronchi. The median interval from initial RT to re-RT was 26.8 (range 11.4–92.3) months. Re-RT was delivered by X-ray beam and proton beam therapy in 20 (95 %) patients and 1 (5 %) patient, respectively. The median radiation dose of re-RT was 60 (range 54–87.5) Gy_10_ and 50 (range 50.0–87.5) Gy_10_ for patients with NSCLC and SCLC, respectively. Grade 3 acute radiation pneumonitis occurred in only one patient. There were no other serious complications. The median follow-up time was 22.1 (range 2.3–56.4) months. The median local progression-free survival time (LPFS) and overall survival time (OS) were 12.9 (95 % confidence interval (CI): 8.9–27.9) months and 31.4 (95 % CI: 16.9–45.9) months, respectively. Patients receiving ≥ 60 Gy_10_ at re-RT had longer LPFS (*p* = 0.04).

**Conclusions:**

Good safety with longer OS than in previous reports was demonstrated. Re-RT seems to be a promising treatment option. Further study to define the risk-benefit ratios is necessary.

## Background

Lung cancer remains one of the most prevalent and deadliest malignancies worldwide, accounting for around 1.8 million deaths each year [1]. External beam radiation therapy (RT) plays an important role in curative management strategies for both small cell lung cancer (SCLC) and non-small cell lung cancer (NSCLC) [2–6]. In NSCLC patients treated with concurrent chemoradiation to the thorax, 5-year rates of locoregional recurrence approach 30 % [7]. In SCLC patients, locoregional recurrence occurred in 36 % of patients [8]. Disease recurrence is still the dominant cause of death after initial treatment.

Second-line chemotherapy is usually selected even when no distant metastasis is observed. For patients with NSCLC and SCLC, progression-free survival (PFS) is 2–8.5 months and 3.3–3.7 months, and median overall survival (OS) is 5.6–11.5 months and 5.5–6.4 months, respectively [9–17].

Re-irradiation (re-RT) has not been considered first-choice, because high-dose re-RT might cause severe radiation injury. Since Green et al reported the efficacy and safety of re-RT as definitive therapy for intrathoracic disease [18], several studies of re-RT have been reported. However, there are few reports of re-RT with curative intent [19–32]. Additionally, there are few detailed reports about the total dose of initial RT and re-RT for each organ at risk [23–28]. Furthermore, due to the characteristics of recurrent disease, long-term observation of treatment results is difficult. The efficacy and safety of re-RT are unclear. Thus, our institutional experience was retrospectively analyzed to investigate the clinical outcomes, including survival and toxicities.

## Methods

This study was approved by the institutional review board of Shizuoka Cancer Center. Medical records were retrospectively obtained. Patients who were registered in the Radiology Information System in Shizuoka Cancer Center between September 2002 and December 2014 were identified. Patients who fulfilled the following criteria were included: (1) the location of the primary tumor was “lung” or “trachea”; (2) recurrences were confirmed by evidence seen on computerized tomography (CT) and/or positron emission tomography (PET) showing the reappearance or enlargement of the tumor shadow within the previous irradiated volume; (3) the patient received external beam radiotherapy to the chest at least twice; (4) the patient was administered at least 45 Gy each time; (5) there was overlap of the two separate dose distributions for the 80 % dose level each time; and (6) there was no evidence of active metastasis at re-RT.

Records were reviewed for patient and disease characteristics, prior fractionated radiotherapy dosimetric parameters, re-RT dosimetric parameters, toxicities, treatment response, local progression-free survival (LPFS), PFS, and OS. Tumors were re-staged using the Union for International Cancer Control TNM Classification of Malignant Tumors 7th edition (UICC 7th). Previous radiotherapy and re-RT variables reviewed included dates of therapy, prescription dose, graphic dose distributions, planning target volume (PTV) size, and tumor location. The treated part was defined as follows: “proximal” was defined as the distal 2 cm of the trachea, the carina, and the right and left main bronchi. Analysis of dose accumulation was carried out after rigid registration using the medical image merge (MIM) (®) software program (version 6.5, MIM Software Inc., Cleveland, OH), if data were available. All doses were recalculated to an equivalent dose of 2 Gy per fraction (EQD2) with the formula: d*n*((d + α/β)/(2 + α/β)), with d the dose per fraction (Gy) and n the number of fractions. For tumor dose and acute side effects, an α/β value of 10 Gy was used (Gy_10_), and for late effects, an α/β value of 3 Gy was used (Gy_3_) [28]. The administration dosage for organs at risk was defined according to the Radiation Therapy Oncology Group (RTOG) contouring atlas of lung (https://www.rtog.org/CoreLab/ContouringAtlases/LungAtlas.aspx). The severities of acute (within 30 days of ending radiation) and late toxicities were evaluated according to the Common Terminology Criteria for Adverse Events (CTCAE) version 4.0. Treatment response was evaluated according to the response evaluation criteria in solid tumors (RECIST) version 1.1. OS was defined as the interval between the index date and the date of death or censored on the last date of confirmed survival, whichever came first. LPFS and PFS were calculated from the index date to the earliest date of local failure and systemic failure or death, whichever came first. Local failure was defined as recurrence within the re-RT site. The index date was the first day of re-RT. The survival curves were calculated using the Kaplan-Meier method, and the differences between the curves were analyzed using the log-rank test. A univariate significance level of *P* < 0.05 was used for identifying predictors.

## Results

**Fig. 1:**
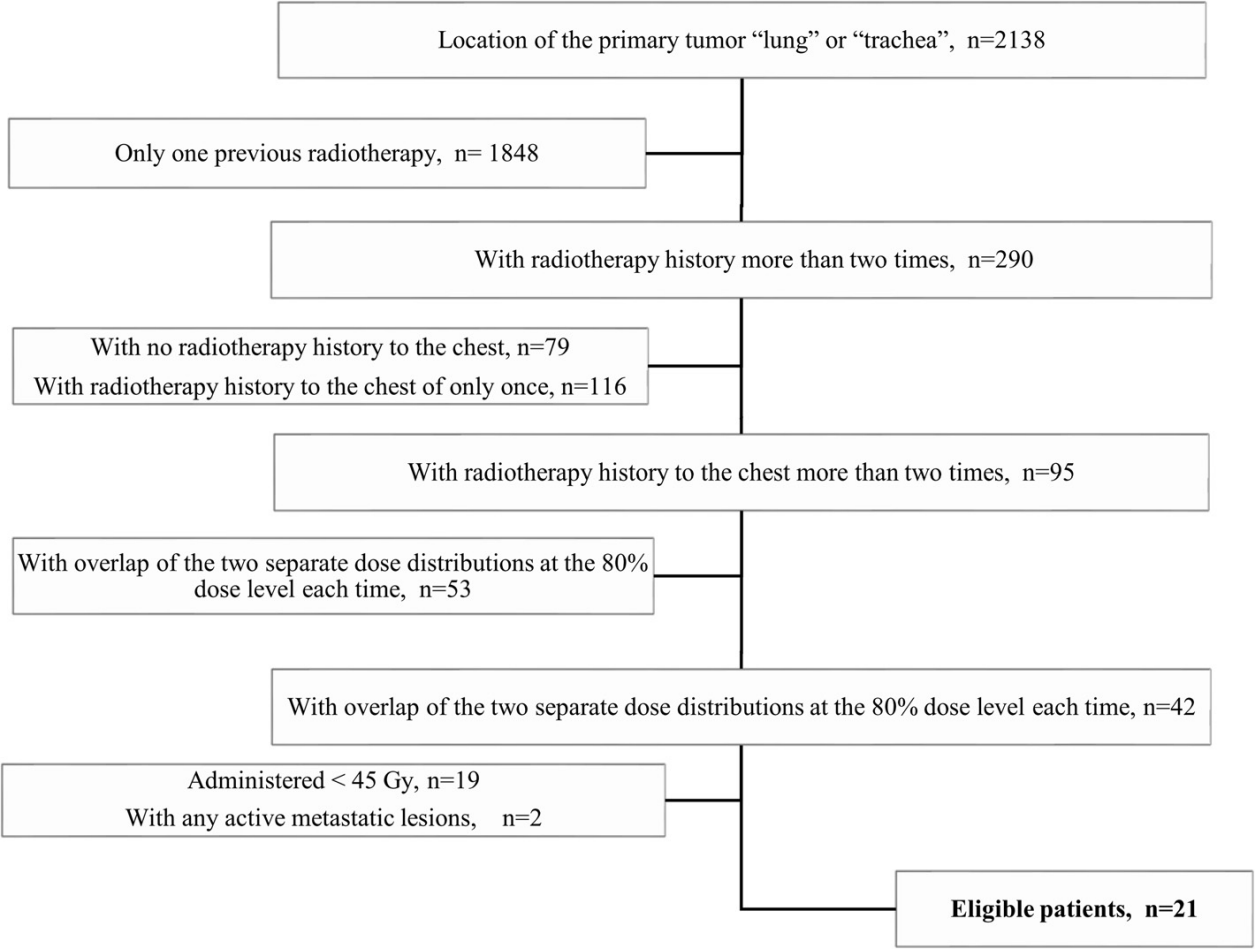
Patient selection and exclusion criteria

Forty-two patients were found to have received re-RT within the previous irradiation volume. Twenty-one patients were excluded; the reasons for exclusion are shown in Fig. 1. Finally, 14 patients (67 %) with NSCLC and seven patients (33 %) with SCLC who received re-RT were identified. Informed consent was obtained from all patients at the time of re-RT.

Patient and disease characteristics at re-RT are shown in Table 1. The median age was 72 years (range 53–85 years). All patients except one had Eastern Cooperative Oncology Group (ECOG) performance status (PS) 0–1. The median interval between the end of initial RT and recurrence was 26.0 months (range 7.0–87.8 months). Recurrence was documented pathologically in only two cases (11 %), and their results were the same as the initial pathology. The majority of tumor recurrence (85 %) occurred at the original tumor site. Fifteen patients (71 %) had proximal recurrent tumor. One patient had stage IV disease at initial RT. He had surgery as initial treatment. He then developed solitary brain metastasis and local tumor recurrence. For both lesions, local radiotherapy appeared to offer the possibility of radical cure.

**Table 1:** Patient and disease characteristics at the time of re-irradiation

Sex, n (%)		
Male	15	(71)
Female	6	(29)
Median age at re-RT, years (range)	72	(53–85)
ECOG PS, n (%)		
0–1	20	(95)
2	1	(5)
Histology at primary RT, n (%)		
NSCLC	14	(67)
Squamous cell carcinoma	6	
Adenocarcinoma	5	
Others	3	
SCLC	7	(33)
Clinical Stage at primary RT, n (%)		
IA / B	4	(19)
IIA / B	4	(19)
IIIA/ B	12	(57)
IV	1	(5)
Pattern of recurrence, n (%)		
Primary tumor	15	(71)
Initially existent metastatic LN (± primary tumor)	3	(14)
Intrapulmonary new lesion	1	(5)
Regional LN new lesion	1	(5)
Both intrapulmonary and regional LN new region	1	(5)
Irradiation tumor location, n (%)		
Proximal	15	(71)
Peripheral	6	(29)

*re-RT* re-irradiation, *PS* performance status, *primary RT* primary radiotherapy, *NSCLC* Non small cell lung cancer, *SCLC* Small cell lung cancer, *LN* lymph node

Initial and re-RT treatment details are shown in Table 2. The median interval from initial RT to re-RT was 26.8 months (range 11.4–92.3 months). At the time of initial RT, the median radiation dose was 60 Gy_10_ (range 51.8–87.5 Gy_10_) for NSCLC patients, while each SCLC patient received 43.1 Gy_10_. Radiotherapy was delivered by X-ray beam therapy in 20 patients (95 %). Only one (5 %) patient with stage IA (UICC 7th) NSCLC refused surgery and was treated using proton beam therapy. He had proximal tumor and chronic heart failure. Proton beam therapy had the advantage of avoiding organs at risk, including the heart. In addition, avoiding organs at risk led to an increased prescription dose. Concurrent chemotherapy was delivered in 16 (76 %) patients. At the time of re-RT, the median radiation dose was 60 Gy_10_ (range 54–87.5 Gy_10_) for NSCLC patients and 50 Gy_10_ (range 50–87.5 Gy_10_) for SCLC patients. Radiotherapy was delivered by X-ray beam therapy in 19 patients (90 %) and by proton beam therapy in 2 patients (10 %). Only one patient (5 %) was administered concurrent chemotherapy. The median PTV volume was 57.4 cc (range 7–601.7 cc). Prior radiotherapy and re-RT details are listed in Table 2.

**Table 2:** Overview of the initial RT and re-RT details

Median interval to re-RT, months (range)			26.8 (11.4–92.3)	
	Initial radiation		Re-irradiation	
Median RT dose, Gy_10_ (range)				
NSCLC	60	(51.8–87.5)	60	(54–87.5)
SCLC	43.1	all patients	50	(50–87.5)
Radiation technique, n (%)				
Conventional fractionation	10	(48)	13	(62)
Hyperfractionation	8	(38)	0	(0)
Hypofractionation	3	(14)	8	(38)
Radiation delivery, n (%)				
Photon therapy	20	(95)	19	(90)
Proton therapy	1	(5)	2	(10)
Concurrent chemotherapy, n (%)				
Yes	16	(76)	1	(5)
No	5	(24)	20	(95)
Median PTV, cc (range)			57.4	(7–601.7)

*RT* radiotherapy, *NSCLC* Non small cell lung cancer, *SCLC* Small cell lung cancer, *PTV* planning target volume

Dose distributions of initial RT and re-RT were accumulated for 13 patients with NSCLC and 5 patients with SCLC. The median cumulative maximum dose of the lung was 130 Gy_3_. The median percentage of total lung volume receiving a cumulative dose of 20 Gy_3_ (V20) was 17 % (range 5–34 %). The mean total lung dose was 12 Gy_3_. The median cumulative dosage that was irradiated to a 1 cc volume and the mean irradiated volume of the organs at risk are shown in Table 3. Four patients received a cumulative dosage of over 50 Gy_3_ to a 1 cc volume.

**Table 3:** Dose distribution of initial RT and re-RT

	Initial	Re-irradiation	Total
Lung dose volume (%)			
V5			
Ipsilateral lung	43	32	55
Contralateral lung	6	0.5	11
Total lung	22	16	30
V20			
Ipsilateral lung	32	15	38
Contralateral lung	2	0	3
Total lung	14	7	17
Mean dose, Gy_3_			
Ipsilateral lung	17	10	25
Contralateral lung	1	1	3
Total lung	8	5	12
Heart	3	7	5
Total median dose, Gy_3_	D1cc	D10cc	
Spinal cord	32	25	
Esophagus	42	33	
Aorta	87	65	
Pulmonary artery	93	61	
Heart	49	46	
Trachea	87	41	
Skin	59	36	
Total mean volume,cc			
Spinal cord ≥40 Gy_3_	0.3		
Spinal cord ≥50 Gy_3_	0		
Heart ≥70 Gy_3_	0		
Esophagus ≥70 Gy_3_	5		
Aorta ≥70 Gy_3_	7		
Pulmonary artery ≥70 Gy_3_	4		

*V5 (20)* The percentage of lung receiving 5 (20) Gy_3_ or more, *OAR* organ at risk, *D1 (10) cc* the dosage irradiated to a 1 (10) cc volume

The most frequent acute toxicity reported was Grade 1–2 radiation dermatitis. No ≥ Grade 3 acute and late toxicities were observed, except in one patient with grade 3 acute pneumonitis that was suspected to be radiotherapy-induced (radiation pneumonitis) (Table 4). Grade 1–2 late pulmonary toxicity was observed in 9 patients (52 %), but there were no serious complications.

**Table 4:** Toxicity

	Grade 1-2	Grade 3	Grade 4
Acute toxicities, n			
Radiation dermatitis	9	0	0
Esophagitis	4	0	0
Pneumonitis	3	1	0
Anorexia	3	0	0
Malaise	2	-	-
Late toxicities, n			
Pneumonitis	9	0	0
Chest wall pain	1	0	-
Pruritus	1	0	-

**Fig. 2:**
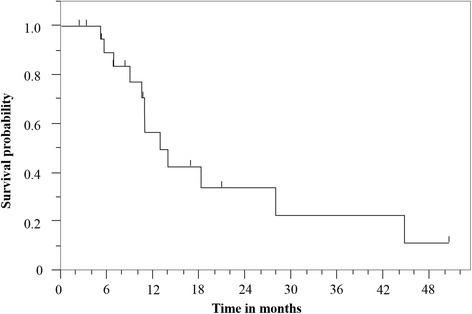
Kaplan-Meier curve of local progression-free survival for all patients

**Fig. 3:**
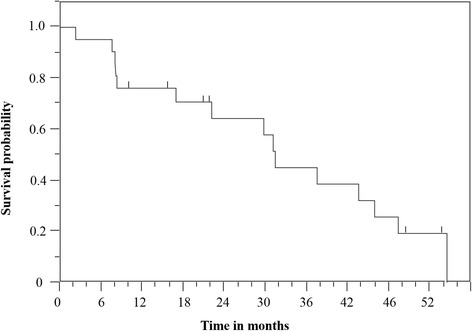
Kaplan-Meier curve of overall survival for all patients

At the time of analysis, 6 patients were alive. The median follow-up time was 22.1 months (range 2.3–56.4 months). The responses to re-RT were complete response (CR) in 4 patients (19 %) and partial response (PR) in 5 patients (24 %). Disease progression after re-RT was observed in 14 patients (67 %). The first failure after re-RT occurred in the field of re-RT in 10 patients (71 %), including 2 patients who had both in and out of field recurrence. Chemotherapy after disease progression was administered to 5 patients (24 %). Among them, three patients had NSCLC, and two patients had SCLC. The median PFS was 10.9 months (95 % confidence interval (CI): 6.8–27.9 months). The median LPFS was 12.9 months (95 % CI: 8.9–27.9 months). The 1-year and 2-year LPFS rates were 57 % and 34 %, respectively (Fig. 2). The median OS was 31.4  months (95 % CI: 16.9–45.9 months). The 1-year and 2-year OS rates were 76 % and 64 %, respectively (Fig. 3).

On univariate analysis, PTV < 75 cc was associated with longer OS (*p* = 0.03), and patients receiving ≥ 60 Gy_10_ at re-RT had longer LPFS (*p* = 0.04). There were no significant differences in OS and LPFS with any other factors. The median OS with or without subsequent chemotherapy was 37.4 months and 31.4 months, respectively; there was no significant difference (*P* = 0.57) (Table 5).

**Table 5:** Univariate analysis: Impact on survival after re-RT

Variable	Number	Median LPFS	*P* value	Median OS	*P* value
Sex					
Male	15	12.9	0.86	31.4	0.12
Female	6	12.4		43.0	
Age, years					
≥ 75	9	13.9	0.96	37.3	0.97
< 75	12	10.9		31.4	
Interval between end of initial RT and recurrence, months					
≥ 24	11	13.8	0.66	31.4	0.48
< 24	10	10.9		31.0	
Interval between initial RT and re-RT, months					
≥ 24	11	13.8	0.66	31.4	0.48
< 24	10	10.9		31.0	
Re-RT dose, Gy_10_					
≥ 60	16	18.2	**0.04**	37.4	0.41
< 60	5	6.8		22.1	
PTV size, cc					
> 75	8	12.9	0.78	16.9	**0.03**
≤ 75	13	13.8		43.5	
Pathology					
NSCLC	14	13.9	0.88	31.4	0.56
SCLC	7	10.9		37.5	
Location					
Proximal	15	2.8	0.62	22.1	0.99
Peripheral	6	18.2		37.4	
Further chemotherapy after re-RT					
Yes	5			37.4	0.57
No	9			31.4	

*RT* radiotherapy, *re-RT* re-irradiation, *NSCLC* Non small cell lung cancer, *SCLC* Small cell lung cancer, *PTV* planning target volume; Bold values represent statistically significant factors

## Discussion

The outcomes of 21 patients given re-RT for recurrent tumors of the thorax were reviewed. There was some therapeutic efficacy with a median follow-up time of 22.1 months and median OS of 31.4 months. To the best of our knowledge, this study had longer follow-up and the best survival outcomes compared with any previous reports regarding re-RT for recurrent lung cancer. In the present study, PTV volume was significantly associated with OS. Reyngold et al. also reported that PTV volume <75 cc was significantly associated with longer OS [29]. The present patients had a generally small PTV. As size increases, the tumor becomes more radiation-resistant due to the presence of hypoxic cells. The generally smaller PTV might have had an impact on both LPFS and OS. It was not possible to identify any other factors significantly associated with OS. However, the good OS might be explained by patient selection. In our institution, we discuss the indications for treatment within a cancer board consisting of thoracic surgeons, thoracic physicians, radiation oncologists, and radiologists. All patients had good ECOG PS of 0-1. The median interval between initial RT and re-RT was 25 months, and all patients except one had an interval over 1 year. Despite a median interval of over 2 years, the majority of tumor recurrence occurred at the original tumor site, with no widening to new regional lymph nodes. This suggests the possibility that the improvement of OS reflected tumors with slow growth and spread. Small PTV volume [29] and long treatment interval [30, 31] are each reported as factors significantly associated with longer OS. This supports the present considerations. Finally, when the present OS is compared with previous reports, with advancing diagnostic technology, it is possible that patients with minute metastases were excluded. In contrast, the median LPFS was not as good as OS. An explanation for this is that the median OS in previous reports was generally poor. There is a possibility that they could not observe recurrence over a sufficient time period in the previous reports. The present result may also reflect longer follow-up. Thus, this does not imply that treatment efficacy was poor. However, receiving a dose over 60 Gy_10_ was significantly associated with longer LPFS. In addition, De Bari et al. also reported that outcome results of re-RT with stereotactic ablative radiotherapy appear superior to those achievable with conventional radiotherapy and/or chemoradiotherapy [22]. Dose escalation needs to be considered.

Grade 3 acute radiation pneumonitis occurred in only one patient. There were no other serious complications. The present results suggest the safety of re-RT over a long period. In addition, it is important that tumor location be reviewed when we think about the safety of radiotherapy to the lung. It has been proposed that the risk of irradiation is greater to centrally located lung tumors even in primary treatment [33]. In the situation of re-RT, this risk may increase. In fact, Trovo et al., Kilburn et al., and Paulen et al. reported fatal toxicity of re-RT for centrally located tumors [22–24, 32]. The present study included centrally located tumors in two-thirds of patients. Despite this, excellent safety was seen with long-term observation. The explanations for the good safety may be as follows. First, the patients had a long interval between primary RT and re-RT. This may contribute to organ repair [34]. Next, the present patients had a generally small PTV volume, which contributed to small irradiation field size. Third, the proportion of patients receiving 2 Gy per fraction was high. This contributed to lower summed EQD2. However, De Ruysscher et al. reported that the incidence of severe lung toxic effects did not differ greatly in patients treated with conventional 3D conformal radiotherapy compared with patients treated with SABR [20]. One of the reasons for this is that the choice of the treatment in studies that they reviewed may have depended on tumor location. Finally, priority was given to avoiding including the spinal cord and esophagus in the irradiation field.

Although patient selection may be required, good OS and safety of re-RT to the thorax were reported. This is beneficial in offering a treatment option to patients with recurrent thoracic tumors. A future problem is to define the risk-benefit ratios with dose escalation.

The present study has several limitations. First, this study was a single-institution, retrospective study. Second, re-RT was not compared with other treatment options for patients with locally recurrent tumor. It is unclear whether re-RT is the optimal treatment for these patients. Third, the number of patients was small, which may explain why statistical significance was not reached. Finally, interval time contributing to the effect of dosage was not considered when the prior and re-RT doses were estimated. When we use the therapeutic procedure in patients with a shorter interval between primary RT and re-RT, attention should be paid to treatment safety, since this has not been established for such patients.

## Conclusions

Re-RT to the thorax for recurrent tumor seems to be a promising and safe treatment option even in patients with proximal tumor. However, local control was not satisfactory. With carefully selected indications, further study is needed to define the optimal treatment dose.

## Abbreviations

CI, confidence interval; CR, complete response; CT, computerized tomography; CTCAE, common terminology criteria for adverse event; ECOG, eastern cooperative oncology group; EQD2, equivalent dose of 2 Gy per fraction; LN, lymph node; LPFS, local progression-free survival; NSCLC, non small cell lung cancer; OS, overall survival; PET, positron emission tomography; PFS, progression-free survival; PR, partial response; PTV, planning target volume; RECIST, response evaluation criteria in solid tumors; re-RT, re-irradiation; RT, radiotherapy; RTOG, radiation therapy oncology group; SABR, stereotactic ablative radiation therapy; SCLC, small cell lung cancer; UICC 7th, the union for international cancer control TNM classification of malignant tumors 7th edition; V20, the median percentages of the lungs receiving a cumulative dose of 20 Gy_3_
